# Supplementary material for: Hexadecenoic Fatty Acid Positional Isomers and De Novo PUFA Synthesis in Colon Cancer Cells
Source: Int J Mol Sci. 2019 Feb 15;20(4):832. doi: 10.3390/ijms20040832 (PMC6412212; doi:10.3390/ijms20040832)
Supplement: Supplementary file 1 [file ijms-20-00832-s001.pdf]

Supplementary Information

Hexadecenoic Fatty Acid Positional Isomers and De Novo PUFA Synthesis in Colon Cancer Cells

Roberta Scanferlato[1,†], Massimo Bortolotti[2,†], Anna Sansone[1], Chryssostomos Chatgilialoglu[1], Letizia Polito[2], Marco De Spirito[3,4], Giuseppe Maulucci[3,4], Andrea Bolognesi[2,‡] and Carla Ferreri[1,‡,*]

[1]Consiglio Nazionale delle Ricerche, ISOF, Area della Ricerca, 40129 Bologna, Italy; r.scanferlato@hotmail.com (R.S.); anna.sansone@isof.cnr.it (A.S.); chrys@isof.cnr.it (C.C.)

[2]Department of Experimental, Diagnostic and Specialty Medicine-DIMES, Alma Mater Studiorum, University of Bologna, 40126 Bologna, Italy; massimo.bortolotti2@unibo.it (M.B.); letizia.polito@unibo.it (L.P.); andrea.bolognesi@unibo.it (A.B.)

[3]Istituto di Fisica, Fondazione Policlinico Universitario A.Gemelli IRCSS, 00168 Roma, Italy; marco.despirito@unicatt.it (M.D.S.); giuseppe.maulucci@unicatt.it (G.M.)

[4]Istituto di Fisica, Università Cattolica del Sacro Cuore, 00168 Roma, Italy

Corresponding author: *Carla Ferreri; email: carla.ferreri@isof.cnr.it

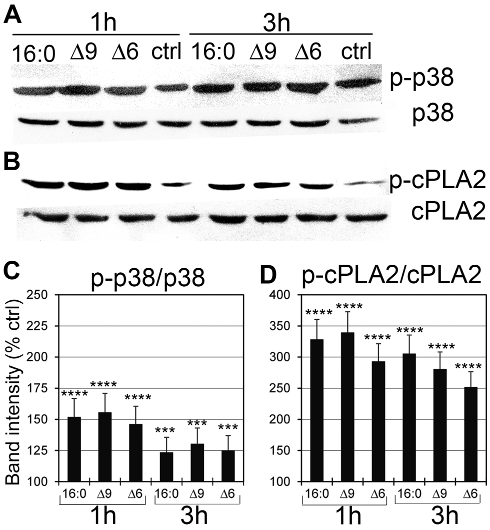

Figure S1. Western blot analyses of p38 (A) and cPLA2 (B) total proteins and phosphorylated forms in Caco-2 cells after supplementation with palmitic (16:0), palmitoleic (Δ9) and sapienic acid (Δ6). Cells were treated for 1 h and 3 h with 150 µM 16:0, 250 µM Δ9 (palmitoleic acid) and 300 µM Δ6 (sapienic acid). Cell lysates were resolved by SDS-PAGE and proteins were blotted and detected with monoclonal antibodies against p38, p-p38, cPLA2 and p-cPLA2. Representative gels are shown. The bar graphs (C and D) represent the band intensity values obtained by Image J analysis, expressed as percentage of the corresponding control. S.D. never exceeds 15% (***, $p<0.001$; ****, $p<0.0001$).

S1 Protocol. Gas chromatographic calibration procedures and quantitative analysis

An internal standard of C17:0 was added in the FAME samples at known concentration. 1 µL of the FAME mixture in *n*-hexane solution was injected in the GC equipment using the split mode (50:1) following the run conditions reported in the text. The results of the Caco-2 cell FAME as control and after treatment were expressed as µg/mL (mean± S.E.M).

Here we reported the LOD (Limit of detection) and LOQ (Limit of quantification) values of the GC instrument used in this work, referred to 8*cis*-18:1 and 5*cis*,8*cis*-18:2 preparing a solution of 1 mg/mL of each standard diluted to a series of appropriate concentrations with *n*-hexane: 8*cis*-18:1, LOD: 0.0008 µg/mL, LOQ: 0.0025 µg/mL; 5*cis*,8*cis*-18:2 LOD 0.0009 µg/mL, LOQ 0.0027 µg/mL.

Table S1. Membrane phospholipid fatty acids of Caco- 2 cells expressed as μg/mL (mean ±S.E.M.). The cells were treated with 150 μM palmitoleic acid and processed at the indicated times. The statistics was carried out in comparison with controls grown under the same conditions without fatty acid.

FAME[1]	Control (n=6) μg/mL	0.5 h (n=3) μg/mL	1h (n=3) μg/mL	3h (n=3) μg/mL
14:0	25.6±1.8	26.1±5.9	20.1±6.4	17.9±1.8*
16:0	393.3±14.6	494.5±23.0*	542.6±105.9	414.5±6.4
6*trans*-16:1	1.8±0.4	2.5±0.9	1.1±0.1	0.2±0.0*
9*trans*-16:1	0.4±0.1	1.9±0.1**	0.44±0.1*	0.5±0.1
6*cis*-16:1	27.0±3.5	18.4±4.4	15.4±3.8	14.9±1.3*
9*cis*-16:1	116.7±4.4	251.3±32.6**	187.1±51.1	344.8±15.2***
18:0	148.5±9.0	135.9±13.0	113.7±2.6*	111.3±27.5
9*trans*-18:1	0.7±0.1	3.1±1.2	0.7±0.1	1.0±0.1
8*cis*-18:1	2.5±0.4	14.7±5.1	1.3±0.1*	1.7±0.2
9*cis*-18:1	391.7±68.3	277.1±117.5	500.0±43.6	407.1±12.6
11*cis*-18:1	216.5±45.9	147.3±12.7	112.0±39.1	166.9±2.4
5*cis*,8*cis* 18:2	8.0±0.7	8.0±0.3	3.5±3.1	2.3±0.3**
18:2 ω 6	50.9±6.5	59.1±8.2	62.5±17.3	38.1±5.7
20:0	1.2±0.3	9.4±1.1**	2.8±1.1	2.7±0.7
20:3 ω6	19.9±2.0	26.8±8.8	3.7±3.2*	2.0±0.3***
20:4 ω6	76.9±4.1	53.4±8.3	33.8±8.6*	47.2±5.8*
20:5 ω3	12.7±4.0	13.1±4.0	7.8±1.8	11.2±2.6
22:0	6.1±2.3	24.1±13.5	1.2±0.7	16.7±2.9*
22:5 ω3	31.0±2.5	26.6±2.9	8.7±3.8**	16.2±2.1*
22:6 ω3	61.843±8.6	45.7±7.6	31.0±13.5	31.5±1.0*
SFA	574.7±25.8	690.0±54.7	680.4±95.2	563.2±30.6
MUFA	754.3±28.6	708.8±64.3	815.7±52.5	935.5±29.6*
PUFA	314.2±36.8	232.8±12.0	151.1±43.9*	148.5±2.5*
ω6	147.7±3.0	139.4±3.4	100.0±29.1	87.3±1.8***
ω3	105.6±12.8	85.5±13.8	47.5±11.8*	58.9±1.2*
Total *trans*	5.4±1.5	7.9±5.4	3.0±0.7	2.4±0.3

[1]FAME identified by the standard references and quantified as described in Materials and Methods; the values are obtained from the GC peak areas (>97% of the total peak areas of the chromatogram recognized by appropriate references). Details of the statistical analysis are reported in Materials and Methods. The statistical significance is estimated on the Standard Error of the Mean;* p value ≤0.049;** p value ≤0.009;*** p value ≤0.0001.

Table S2. Membrane phospholipid fatty acids of Caco- 2 cells expressed as μg/mL (mean ±S.E.M.). The cells were treated with 300 μM palmitoleic acid and processed at the indicated times. The statistics was carried out in comparison with controls grown without supplementation for the same time intervals.

FAME[1]	Control (n=6) μg/mL	0.5 h (n=3) μg/mL	1h (n=3) μg/mL	3h (n=3) μg/mL
14:0	25.6±1.8	10.7±1.4*	24.1±2.6	26.1±15.1
16:0	393.3±14.6	355.9±30.2	370.6±37.6	410.7±72.7
6*trans*-16:1	1.8±0.4	2.7±0.5	2.4±0.4	1.1±0.4
9trans-16:1	0.4±0.1	2.7±0.5*	2.3±0.3**	1.1±0.4
6cis-16:1	27.0±3.5	14.6±2.7*	18.2±3.6	9.6±3.0*
9cis-16:1	116.7±4.4	313.0±51.5**	381.0±49.2**	369.1±74.6*
18:0	148.5±9.0	124.1±17.4	124.5±23.1	102.6±16.3
9trans-18:1	0.7±0.1	4.9±2.6	1.7±0.6	3.5±1.2
8cis-18:1	2.5±0.4	4.9±1.0	9.3±3.4	5.9±2.1
9cis-18:1	391.7±68.3	410.8±11.9	323.8±33.2	418.9±59.2
11cis-18:1	216.5±45.9	159.2±7.2	129.6±16.58	124.9±17.3
5cis,8cis-18:2	8.0±0.7	12.6±2.2	12.1±5.8	4.1±1.4
18:2 ω 6	50.9±6.5	83.5±30.2	27.2±5.3*	37.9±8.3
20:0	1.2±0.3	13.4±3.5*	7.6±3.2	24.7±13.3
20:3 ω6	19.9±2.0	24.3±0.4	19.6±11.3	8.9±3.1*
20:4 ω6	76.9±4.1	45.1±6.3*	46.9±3.8**	38.4±5.5**
20:5 ω3	12.7±4.0	8.8±2.0	15.0±1.1	7.1±3.2
22:0	6.1±2.3	9.5±1.8	49.9±30.1	6.7±3.6
22:5 ω3	31.0±2.5	16.0±2.5*	19.4±3.1*	20.2±4.9
22:6 ω3	61.8±8.6	26.2±4.0*	58.3±9.0	25.7±7.1*
SFA	574.7±25.8	513.4±46.2	576.8±77.4	574.1±60.1
MUFA	754.3±28.6	901.8±44.2*	861.9±98.9	925.0±41.8*
PUFA	314.2±36.8	216.6±21.2	198.7±20.8	142.4±29.5*
ω6	147.7±3.0	152.9±25.4	93.8±15.4*	85.2±16.0*
ω3	105.6±12.8	51.0±7.8*	119.4±26.5	53.1±12.5*
Total *trans*	5.4±1.5	17.4±4.1*	12.1±3.1	8.1±2.9

[1]FAME identified by the standard references and quantified as described in Materials and Methods; the values are obtained from the main GC peak areas (>97% of the total peak areas of the chromatogram). Details of the statistical analysis are reported in Materials and Methods. The statistical significance is estimated on Standard Error of the Mean. * p value ≤0.049; ** p value ≤0.0096; *** p value ≤0.0009

Table S3. Membrane phospholipid fatty acids of Caco- 2 cells expressed as µg/mL (mean ±S.E.M.). The cells were treated with 150 µM sapienic acid and processed at the indicated times. The statistics was carried out in comparison with controls grown without supplementation for the same time intervals.

FAME[1]	Control (n=6) µg/mL	0.5 h (n=3) µg/mL	1h (n=3) µg/mL	3h (n=3) µg/mL
14:0	25.6±1.8	21.0±2.9	20.2±2.1	20.1±0.3*
16:0	393.3±14.6	361.2±60.7	327.6±5.2*	303.2±20.0*
6*trans*-16:1	1.8±0.4	2.4±0.8	2.0±0.1	10.4±7.1
9trans-16:1	0.4±0.1	1.3±0.3	0.7±0.2	4.9±4.0
6cis-16:1	27.0±3.5	57.8±20.8	209.2±1.3***	247.1±10.7***
9cis-16:1	116.7±4.4	141.2±55.1	90.5±1.1**	117.4±4.8
18:0	148.5±9.0	209.0±55.7	134.1±3.7	120.0±2.9*
9trans-18:1	0.7±0.1	3.3±1.3	1.4±0.3	3.6±0.8*
8cis-18:1	2.5±0.4	21.9±4.4*	26.2±3.9**	102.8±11.7**
*9cis*18:1	391.7±68.3	335.4±31.3	380.4±11.1	332.2±8.0
11cis-18:1	216.5±45.9	158.3±5.9	148.3±3.0	151.5±7.7
5cis,8cis-18:2	8.0±0.7	16.7±5.6	12.9±0.3**	13.1±0.3**
18:2 ω 6	50.9±6.5	60.0±3.9	37.6±0.7	26.9±1.4*
20:0	1.2±0.3	9.3±0.5***	2.7±0.3*	12.3±5.6
20:3 ω6	19.9±2.0	28.7±3.0	22.3±1.5	23.0±5.2
20:4 ω6	76.9±4.1	84.0±15.5	96.2±5.4*	59.4±2.6*
20:5 ω3	12.7±4.0	15.2±1.1	17.9±1.4	13.9±5.8
22:0	6.1±2.3	13.8±6.5	11.0±1.1	10.1±3.4
22:5 ω3	31.0±2.5	43.2±16.8	38.5±2.9	34.0±7.6
22:6 ω3	61.8±8.6	58.3±17.4	66.7±1.2	36.6±7.1
SFA	574.7±25.8	614.3±36.4	495.7±5.9*	465.8±15.5*
MUFA	754.3±28.6	714.7±19.8	854.5±6.0*	951.1±28.0**
PUFA	314.2±36.8	306.1±26.7	292.1±11.3	207.0±28.2*
ω6	147.7±3.0	172.7±16.8	156.1±7.6	109.3±8.6*
ω3	105.6±12.8	116.7±15.6	123.1±4.7	84.5±20.3
Total *trans*	5.4±1.5	14.5±3.2	6.6±0.37	10.0±3.3

[1]FAME identified by the standard references and quantified as described in Materials and Methods; the values are obtained from the main GC peak areas (>97% of the total peak areas of the chromatogram). Details of the statistical analysis are reported in Materials and Methods. The statistical significance is estimated on Standard Error of the Mean. * p value ≤0.049;** p value ≤0.009;*** p value ≤0.0001.

Table S4. Membrane phospholipid fatty acids of Caco- 2 cells expressed as μg/mL (mean ±S.E.M.). The cells were treated with 300 μM sapienic acid and processed at the indicated times. The statistics was carried out in comparison with controls grown without supplementation for the same time intervals.

FAME[1]	Control (n=6) μg/mL	0.5 h (n=3) μg/mL	1h (n=3) μg/mL	3h (n=3) μg/mL
14:0	25.6±1.8	11.1±0.7*	6.3±3.0**	19.7±1.2
16:0	393.3±14.6	388.3±28.0	304.5±29.0*	299.6±0.5**
6*trans*-16:1	1.8±0.4	1.7±0.5	2.2±0.2	1.7±0.5
9trans-16:1	0.4±0.1	1.2±0.1*	0.8±0.1	1.1±0.1*
6cis-16:1	27.0±3.5	137.1±13.0*	208.7±24.2**	417.8±7.9***
9cis-16:1	116.7±4.4	80.6±6.9**	66.2±3.4**	110.8±4.9
18:0	148.5±9.0	157.9±3.8	173.6±8.4	102.9±4.9*
9trans-18:1	0.7±0.1	7.1±4.4	2.0±0.4*	1.6±0.6
8cis-18:1	2.5±0.4	62.0±15.8*	49.5±9.9**	72.3±2.3***
9cis-18:1	391.7±68.3	247.0±101.7	361.1±22.0	305.7±4.7
11cis-18:1	216.5±45.9	202.5±52.4	164.8±14.0	115.4±7.5
5cis,8cis-18:2	8.0±0.7	13.0±0.8**	11.0±1.4	12.2±1.7
18:2 ω 6	50.9±6.5	34.8±11.9	30.2±9.6	28.7±3.2*
20:0	1.2±0.3	19.8±16.7*	2.4±0.6	7.5±1.4*
20:3 ω6	19.9±2.0	22.4±6.8	18.8±1.9	21.7±0.7
20:4 ω6	76.9±4.1	94.4±3.7*	87.2±2.3	60.2±2.5*
20:5 ω3	12.7±4.0	8.4±5.3	11.9±1.7	10.6±2.1
22:0	6.1±2.3	13.8±7.4	11.0±2.5	3.7±2.1
22:5 ω3	31.0±2.5	47.8±6.2*	28.1±1.9	22.7±3.7
22:6 ω3	61.8±8.6	79.6±13.4*	109.0±44.7	31.5±0.8*
SFA	574.7±25.8	549.1±13.5	490.1±30.8	433.5±5.0**
MUFA	754.3±28.6	771.2±4.0*	850.3±21.7	1022.0±10.7***
PUFA	314.2±36.8	300.4±19.5	293.1±51.5	187.6±7.3*
ω6	147.7±3.0	151.6±21.0	133.0±11.2	110.6±1.4***
ω3	105.6±12.8	135.8±14.4*	149.0±45.5	64.8±5.8*
Total *trans*	5.4±1.5	18.8±8.5	8.4±0.9	6.4±0.5

[1]FAME identified by the standard references and quantified as described in Materials and Methods; the values are obtained from the main GC peak areas (>97% of the total peak areas of the chromatogram). Details of the statistical analysis are reported in Materials and Methods. The statistical significance is estimated on Standard Error of the Mean. * p value ≤0.049;** p value ≤0.0096;*** p value ≤0.0009

Table S5. Membrane phospholipid fatty acids of Caco- 2 cells expressed as µg/mL (mean ±S.E.M.). The cells were treated with 150 µM palmitic acid and processed at the indicated times. The statistics was carried out in comparison with controls grown without supplementation for the same time intervals.

FAME[1]	Control (n=6) µg/mL	0.5 h (n=3) µg/mL	1h (n=3) µg/mL	3h (n=3) µg/mL
14:0	25.6±1.8	38.4±6.6**	13.3±1.6	19.7±8.6
16:0	393.3±14.6	497.8±56.2	456.1±42.0	535.1±42.0*
6*trans*-16:1	1.8±0.4	3.3±1.4	10.2±6.4	1.5±0.0
9*trans*-16:1	0.4±0.1	2.4±0.9	6.8±5.1	0.8±0.2
6*cis*-16:1	27.0±3.5	44.8±9.5	20.9±1.5	26.2±4.9
9*cis*-16:1	116.7±4.4	130.7±21.5	114.1±9.5	101.4±41.4
18:0	148.5±9.0	115.6±12.3	155.0±7.9	117.2±1.3*
9*trans*-18:1	0.7±0.1	3.8±0.1***	4.1±2.3	3.8±1.6
8*cis*-18:1	2.5±0.4	2.9±0.8	10.0±5.2	2.0±1.2
9*cis*-18:1	391.7±68.3	405.6±31.5	420.7±9.1	424.5±5.3
11*cis* 18:1	216.5±45.9	167.4±15.3	172.5±4.3	175.3±6.5
5*cis*,8*cis*-18:2	8.0±0.7	3.3±1.9	4.4±1.4	3.5±0.6**
18:2 ω 6	50.9±6.5	35.2±8.0	44.2±3.1	25.7±1.1*
20:0	1.2±0.3	20.6±9.2	8.2±2.3*	4.4±1.9
20:3 ω6	19.9±2.0	12.9±4.0	17.7±3.1	17.1±1.8
20:4 ω6	76.9±4.1	51.3±19.9	65.0±3.8	71.5±9.0
20:5 ω3	12.7±4.0	8.8±1.5	9.7±3.8	12.6±3.0
22:0	6.1±2.3	11.1±4.1	12.6±5.6	5.3±1.0
22:5 ω3	31.0±2.5	36.7±4.5	35.1±3.6	37.3±1.2
22:6 ω3	61.8±8.6	51.3±11.5	63.2±10.3	58.9±9.3
SFA	574.7±25.8	683.6±74.5	645.4±34.5	681.7±49.9
MUFA	754.3±28.6	751.4±59.8	738.3±4.4	729.5±27.7
PUFA	314.2±36.8	199.5±23.7	239.4±15.8	226.6±24.7
ω6	147.7±3.0	99.3±17.4	127.0±6.1*	114.3±11.1*
ω3	105.6±12.8	96.8±15.8	108.0±12.0	108.8±13.5
Total *trans*	5.4±1.5	15.1±3.9	26.5±16.0	9.3±2.2

[1]FAME identified by the standard references and quantified as described in Materials and Methods; the values are obtained from the main GC peak areas (>97% of the total peak areas of the chromatogram). Details of the statistical analysis are reported in Materials and Methods. The statistical significance is estimated on Standard Error of the Mean. * p value ≤0.049; ** p value ≤0.009; *** p value ≤0.0001

Table S6. Membrane phospholipid fatty acids of Caco- 2 cells expressed as µg/mL (mean ±S.E.M.). The cells were treated with 300 µM palmitic acid and processed at the indicated times. The statistics was carried out in comparison with controls grown without supplementation for the same time intervals.

FAME[1]	Control (n=6) µg/mL	0.5 h (n=3) µg/mL	1h (n=3) µg/mL	3h (n=3) µg/mL
14:0	25.6±1.8	24.8±7.0	34.0±8.3	24.4±0.8
16:0	393.3±14.6	521.0±75.2	490.5±27.3*	648.2±26.2**
6*trans*-16:1	1.8±0.4	2.7±0.9	4.2±0.3*	1.9±1.0
9trans-16:1	0.4±0.1	2.1±0.6	2.0±1.0	1.6±0.9
6cis-16:1	27.0±3.5	16.3±3.6*	23.8±8.6	14.8±0.9*
9cis-16:1	116.7±4.4	154.8±33	159.9±36.5	126.3±9.4
18:0	148.5±9.0	140.1±9.9	142.4±11.5	118.8±23.3
9trans-18:1	0.7±0.1	5.6±3.2	4.5±1.3	1.3±0.5*
8cis-18:1	2.5±0.4	9.5±4.5	8.7±2.4	5.0±1.1
9cis-18:1	391.7±68.3	369.2±22.5	293.0±115.8	319.8±9.2
11cis-18:1	216.5±45.9	138.3±7.8	183.3±64.2	115.8±5.6
5cis,8cis-18:2	8.0±0.7	2.7±1.3*	2.7±0.7**	2.6±0.1**
18:2 ω 6	50.9±6.5	80.8±10.4	63.7±37.2	54.5±13.7
20:0	1.2±0.3	18.7±8.8	15.8±4.9*	25.0±10.3
20:3 ω6	19.9±2.0	18.4±5.4	13.4±1.8	9.4±1.6*
20:4 ω6	76.9±4.1	47.9±4.9*	72.3±12.0	57.2±4.1*
20:5 ω3	12.7±4.0	16.2±1.4	16.5±4.6	16.9±2.8
22:0	6.1±2.3	15.3±5.4	17.9±1.8*	29.3±9.3
22:5 ω3	31.0±2.5	23.0±0.9*	32.3±10.9	22.7±0.9*
22:6 ω3	61.8±8.6	34.3±4.4*	61.6±6.5	49.8±3.4
SFA	574.7±25.8	720.0±60.7	700.5±47.5	845.8±23.1**
MUFA	754.3±28.6	688.3±53.2	668.7±75.7	581.7±7.0*
PUFA	314.2±36.8	223.3±11.1	262.5±43.0	213.2±13.8
ω6	147.7±3.0	147.1±10.6	149.4±38.5	121.1±15.9
ω3	105.6±12.8	73.5±5.6	110.4±18.5	89.5±5.3
Total *trans*	5.4±1.5	18.0±7.0	17.7±1.6*	8.8±2.9

[1]FAME identified by the standard references and quantified as described in Materials and Methods; the values are obtained from the main GC peak areas (>97% of the total peak areas of the chromatogram). Details of the statistical analysis are reported in Materials and Methods. The statistical significance is estimated on Standard Error of the Mean. * p value ≤0.049;** p value ≤0.0096;*** p value ≤0.0009.

Table S7. Membrane phospholipid fatty acids of Caco-2 cells expressed as μg/mL(mean ±S.E.M.) treated for 24 h with A (palmitoleic acid 150 μM) or B (sapienic acid 150 μM) or C (palmitic acid 150 μM), compared to controls grown in the same time without supplementation.

FAME[1]	Control (n=6) μg/mL	A (n=3) μg/mL	B (n=3) μg/mL	C (n=3) μg/mL
14:0	26.6±1.6	17.7±2.3*	19.0±2.5	21.7±1.1
16:0	473.6±33.5	396.8±101.2	242.4±15.9**	642.8±11.3**
6trans-16:1	1.4±0.2	0.6±0.1*	2.1±0.5	1.2±0.6
9trans-16:1	1.2±0.2	1.0±0.2	1.0±0.3	1.3±0.7
6cis-16:1	27.5±5.5	11.9±0.3*	256.2±26.3**	24.1±4.5
9cis-16:1	141.7±17.2	245.3±58.0	63.3±4.8*	167.3±16.7
18:0	128.8±10.8	103.0±8.6	150.6±11.5	110.2±2.9
9 trans-18:1	1.8±0.6	2.4±0.5	1.2±0.6	4.7±1.8
8cis-18:1	5.0±1.7	6.7±3.9	327.2±10.5***	9.5±3.1
9cis-18:1	466.6±19.6	450.7±104.6	320.7±89.6	336.5±18.2**
11cis-18:1	173.6±9.8	235.4±70.4	129.1±11.1	172.9±10.8
5cis,8cis-18:2	5.1±1.6	13.3±6.0	16.8±3.2**	3.8±0.7
18:2 ω 6	33.9±0.7	41.0±17.1	29.3±7.2	35.1±6.0
20:0	3.7±2.8	5.5±1.2	3.1±1.8	5.8±1.1
20:3 ω6	18.5±2.7	33.8±8.4	16.0±3.3	15.1±3.2
20:4 ω6	55.2±8.5	28.8±8.5	76.0±7.9	30.8±7.3
20:5 ω3	17.4±6.6	6.5±1.3	12.2±2.8	5.9±2.4
22:0	1.5±0.5	3.9±0.8	8.7±3.9	12.6±8.2
22:5 ω3	25.5±4.1	17.0±0.5	28.8±2.8	13.3±5.6
22:6 ω3	37.4±3.8	24.5±3.7	42.1±6.0	29.6±1.4
SFA	634.1±42.6	527.0±107.3	423.8±8.0*	793.2±16.2*
MUFA	814.5±53.5	950.0±107.2	1096.5±86.0	710.4±16.5
PUFA	193.1±11.6	165.1±6.5	221.3±23.9	133.7±12.1
ω6	107.7±9.2	103.6±15.7	121.4±17.9	81.0±6.0*
ω3	80.3±1.4	48.1±5.6*	83.0±9.2	48.9±7.1*
Total *trans*	7.9±0.7	10.6±4.6	7.9±1.0	12.3±1.9

[1]FAME identified by the standard references and quantified as described in Materials and Methods; the values are obtained from the main GC peak areas (>97% of the total peak areas of the chromatogram). Details of the statistical analysis are reported in Materials and Methods. The statistical significance is estimated on Standard Error of the Mean. * p value ≤0.049;** p value ≤0.009;*** p value ≤0.0001

Table S8. Membrane phospholipid fatty acids of Caco-2 cells expressed as μg/mL(mean ±S.E.M.) treated for 24 h with A (palmitoleic acid 300 μM) or B (sapienic acid 300 μM) or C (palmitic acid 300 μM), compared to controls grown in the same time without supplementation.

FAME[1]	Control (n=6) μg/mL	A (n=3) μg/mL	B (n=3) μg/mL	C (n=3) μg/mL
14:0	26.6±1.6	11.8±2.6**	18.8±0.5*	21.0±7.8
16:0	473.6±33.5	263.3±28.9**	276.8±13.1**	777.4±81.3*
6trans-16:1	1.4±0.2	0.8±0.3	2.5±0.4	0.5±0.1*
9trans-16:1	1.2±0.2	0.7±0.3	2.3±0.5	0.7±0.1
6cis-16:1	27.5±5.5	7.3±1.2*	397.5±10.1***	11.9±4.6
9cis-16:1	141.7±17.2	357.6±113.2	56.4±1.8**	86.4±22.9
18:0	128.8±10.8	87.0±1.0*	115.4±1.6	179.8±41.3
9trans-18:1	1.8±0.6	2.5±1.0	4.3±1.5	1.6±0.4
8cis-18:1	5.0±1.7	6.0±1.6	268.7±22.5***	3.7±1.2
9cis-18:1	466.6±19.6	505.1±167.9	209.1±24.4**	384.8±108.4
11cis-18:1	173.6±9.8	229.4±17.3*	79.3±5.8**	62.7±13.1**
5cis,8cis-18:2	5.1±1.6	3.7±1.0	15.4±1.2**	1.6±0.5
18:2 ω 6	33.9±0.7	55.0±13.3	27.3±2.0*	35.7±7.4
20:0	3.7±2.8	29.1±15.3	12.2±0.5*	13.8±2.1*
20:3 ω6	18.5±2.7	12.0±4.2	14.8±4.8	5.1±1.0**
20:4 ω6	55.2±8.5	28.8±6.3	69.9±5.8	30.2±0.6*
20:5 ω3	17.4±6.6	6.6±2.3	11.6±3.1	3.0±0.5
22:0	1.5±0.5	7.3±3.0	11.1±2.3*	5.7±0.8*
22:5 ω3	25.5±4.1	13.9±2.3	24.6±2.2	11.6±3.0
22:6 ω3	37.4±3.8	19.2±4.6*	26.5±2.1	10.9±3.0**
SFA	634.1±42.6	398.5±45.6*	434.4±14.6*	997.8±50.2**
MUFA	814.5±53.5	1105.4±38.2*	1010.9±5.5*	549.5±66.8*
PUFA	193.1±11.6	139.2±18.0	190.2±12.3	98.2±15.8**
ω6	107.7±9.2	95.8±10.3	112.0±6.6	71.1±9.0*
ω3	80.3±1.4	39.7±7.9**	62.8±7.4	25.5±6.5**
Total *trans*	7.9±0.7	6.5±2.0	14.1±1.0**	4.0±1.0*

[1]FAME identified by the standard references and quantified as described in Materials and Methods; the values are obtained from the main GC peak areas (>97% of the total peak areas of the chromatogram). Details of the statistical analysis are reported in Materials and Methods. The statistical significance is estimated on Standard Error of the Mean. * p value ≤0.049; ** p value ≤0.0096; *** p value ≤0.0009

Table S9. Triglyceride fatty acids of Caco-2 cells expressed as μg/mL (mean ±S.E.M.) treated for 24 hours with 150μM (A), 300 μM (B) palmitoleic acid and 150μM (C),300 μM (D) palmitic acid

FAME[1]	A (n=3) μg/mL	B (n=3) μg/mL	C (n=3) μg/mL	D (n=3) μg/mL
14:0	7.4±1.2	4.1±0.6	4.3±1.1	6.0±1.1
16:0	80.2±4.9	60.3±16.7	143.9±27.8	258.7±31.4
6*trans*-16:1	0.2±0.2	n.d.	0.1±0.1	0.2±0.1
9trans-16:1	0.1±0.1	n.d.	0.3±0.1	0.3±0.2
6cis-16:1	2.4±0.5	2.1±0.8	3.4±1.8	3.5±0.9
9cis-16:1	103.9±10.5	147.6±37.6	34.6±19.7	23.1±13.0
18:0	24.6±1.5	21.3±10.2	45.3±12.1	35.5±4.5
9trans-18:1	0.3±0.3	n.d.	0.3±0.2	0.5±0.1
8cis-18:1	0.5±0.5	0.5±0.5	2.1±0.4	0.9±0.4
9cis-18:1	60.8±1.5	101.9±45.9	115.9±27.1	64.2±8.2
11cis-18:1	131.9±17.5	99.7±22.9	47.4±3.4	38.3±8.7
5cis,8cis-18:2	1.0±0.2	0.1±0.1	1.5±0.7	0.5±0.2
18:2 ω 6	26.5±16.2	14.5±5.2	6.6±3.4	6.8±2.7
20:0	2.8±1.1	0.6±0.1	5.9±4.2	2.6±1.1
20:3 ω6	2.9±0.8	0.8±0.2	4.4±1.2	3.8±0.9
20:4 ω6	3.3±1.0	2.7±1.0	22.7±12.0	5.0±0.7
20:5 ω3	2.5±0.5	1.0±0.4	6.9±3.8	1.6±0.2
22:0	2.1±0.4	0.6±0.2	3.2±0.6	1.3±0.3
22:5 ω3	2.3±0.8	1.1±0.1	3.7±1.8	3.3±0.8
22:6 ω3	4.5±0.7	1.7±0.2	7.7±2.9	4.1±1.8
SFA	117.2±8.2	87.0±26.4	202.7±26.4	304.2±34.6
MUFA	299.5±8.0	351.9±30.5	203.5±28.6	130.0±30.1
PUFA	43.0±14.9	22.0±5.1	53.4±10.3	25.2±5.1
ω6	32.8±14.7	18.1±5.4	33.6±14.8	15.6±2.4
ω3	9.3±0.7	3.8±0.3	18.3±5.3	9.1±2.9
Total *trans*	1.3±1.3	0.1±0.1	1.5±0.8	1.6±0.2

[1]FAME identified by the standard references and quantified as described in Materials and Methods; the values are obtained from the main GC peak areas (>97% of the total peak areas of the chromatogram); nd: not detected.

Table S10. Triglyceride fatty acids of Caco-2 cells expressed as µg/mL(mean ±S.E.M.) treated for the indicated times with 150 µM sapienic acid.

FAME[1]	0.5 h (n=3) µg /mL	1h (n=3) µg /mL	3h (n=3) µg /mL	24h (n=3) µg /mL
14:0	4.4±0.1	2.9±0.1	9.2±0.2	4.1±0.1
16:0	72.2 ± 0.7	47.7±1.1	77.6±1.3	76.9±0.3
6*trans*-16:1	n.d.	n.d.	n.d.	n.d.
9trans-16:1	n.d.	n.d.	n.d.	n.d.
6cis-16:1	72.8±1.0	174.9±1.5	111.9±1.8	62.1±0.3
9cis-16:1	13.4 ± 0.2	8.4±0.2	15.0±0.7	15.1±0.3
18:0	30.1±1.2	17.5±0.6	35.0±0.9	40.4±0.7
9trans-18:1	n.d.	0.3±0.1	0.3±0.3	n.d.
8cis-18:1	21.1±1.3	10.9±0.7	18.2±2.2	57.9±7.7
9cis-18:1	156.1±6.3	138.5±4.0	108.5±4.8	100.8±7.5
11cis-18:1	53.0±1.4	24.7±1.6	38.5±4.5	40.4±1.4
5cis,8cis-18:2	2.7±0.3	2.7±0.1	8.6±2.2	4.4±1.1
18:2 ω 6	8.2±0.5	8.7±0.4	8.4±0.6	9.7±0.2
20:0	0.9±0.0	1.8±0.3	3.2±0.9	4.7±1.5
20:3 ω6	3.1±0.2	2.7±0.9	2.6±0.1	4.4±1.1
20:4 ω6	4.1±0.2	3.8±1.5	6.8±0.6	8.6±0.1
20:5 ω3	1.7±0.2	1.2±0.1	2.5±0.6	4.0±0.5
22:0	0.7±0.1	0.7±0.1	2.4±0.9	3.3±0.6
22:5 ω3	2.6±1.1	3.3±0.9	4.3±0.5	7.2±1.4
22:6 ω3	13.8±1.4	10.4±1.8	7.8±0.9	16.9±1.2
SFA	108.3±1.5	70.5±1.1	127.4±2.2	129.4±1.4
MUFA	316.5±4.1	357.4±4.1	292.2±1.7	276.3±1.1
PUFA	36.3±2.7	32.8±4.5	40.9±2.5	55.4±1.9
ω6	15.4±0.6	15.2±2.0	17.8±0.1	22.8±0.9
ω3	18.2±2.5	14.9±2.5	14.6±0.5	28.1±1.5
Total *trans*	n.d.	0.3±0.1	0.5±0.5	n.d.

[1]FAME identified by the standard references and quantified as described in Materials and Methods; the values are obtained from the main GC peak areas (>97% of the total peak areas of the chromatogram); nd= not detected.

Table S11. Triglyceride fatty acids of Caco-2 cells expressed as µg/mL(mean ±S.E.M.) treated for the indicated times with 300 µM sapienic acid.

FAME[1]	0.5 h (n=3) µg/mL	1h (n=3) µg/mL	3h (n=3) µg/mL	24h (n=3) µg/mL
14:0	4.0±0.6	7.4±0.7	16.4±0.8	19.1±1.1
16:0	44.9±1.1	63.2±0.1	49.6±2.4	156.7±22.4
6*trans*-16:1	0.9±0.2	1.0±1.0	n.d.	n.d.
9trans-16:1	0.1±0.1	n.d.	n.d.	n.d.
6cis-16:1	154.0±2.7	212.2±2.2	199.8±9.3	42.6±4.6
9cis-16:1	10.3±0.3	8.1±1.1	15.1±1.6	16.8±0.8
18:0	75.1±0.7	26.0±2.2	27.1±2.4	57.2±5.9
9trans-18:1	0.2±0.1	n.d.	n.d.	n.d.
8cis-18:1	12.9±2.7	22.3±4.0	22.5±5.4	20.8±2.3
9cis-18:1	65.6±5.3	67.6±6.0	39.4±6.3	71.2±5.2
11cis-18:1	33.8±2.8	14.8±6.1	22.3±7.0	24.6±1.1
5cis,8cis-18:2	2.1±0.8	2.1±0.1	11.1±3.2	5.4±1.1
18:2 ω 6	15.6±0.8	8.1±2.9	9.6±2.2	8.8±1.1
20:0	1.3±0.3	1.9±0.6	3.9±0.8	4.7±0.5
20:3 ω6	2.7±0.8	3.3±0.8	3.3±0.6	5.0±1.8
20:4 ω6	8.2±0.2	4.7±1.9	11.4±5.6	7.7±1.6
20:5 ω3	1.5±0.5	2.2±0.3	7.8±3.1	4.7±1.0
22:0	1.1±0.3	1.2±0.3	4.7±1.6	2.9±0.5
22:5 ω3	3.8±0.6	3.4±1.2	5.1±1.6	5.4±1.6
22:6 ω3	22.2±1.3	11.3±3.2	11.7±0.7	7.4±1.2
SFA	126.4±0.8	99.7±2.1	101.8±0.8	240.5±17.1
MUFA	276.6±4.4	325.1±11.6	299.2±4.2	176.0±11.7
PUFA	56.3±3.7	35.2±8.5	60.1±4.3	44.5±7.3
ω6	26.6±1.3	16.1±5.6	24.3±5.3	21.5±4.3
ω3	27.6±2.2	16.9±3.9	24.6±5.2	17.6±2.0
Total *trans*	1.8±0.3	1.0±1.0	n.d.	n.d.

[1]FAME identified by the standard references and quantified as described in Materials and methods; the values are obtained from the main GC peak areas (>97% of the total peak areas of the chromatogram); nd = not detected.

Table S12. Cholesteryl ester fatty acids of Caco-2 cells expressed as μg/mL(mean ±S.E.M.) formed after treatment for 1 hour and 3 hours with 150 μM sapienic acid, and after 0.5, 1 and 3 hours with 300 μM sapienic acid.

FAME[1]	1h 150μM (n=3)μg/mL	3h 150μM (n=3) μg/mL	0.5h 300μM (n=3) μg/mL	1h 300μM (n=3) μg/mL	3h 300μM (n=3) μg/mL
14:0	8.3±1.5	2.2±0.3	6.1±0.3	4.5±2.1	6.1±0.1
16:0	66.1±0.7	52.3±1.1	62.3±0.7	62.8±1.8	71.1±1.2
6*trans*-16:1	n.d.	n.d.	0.1±0.1	0.2±0.2	0.1±0.1
9trans-16:1	n.d.	n.d.	n.d.	n.d.	n.d.
6cis-16:1	10.0±6.4	19.1±4.1	16.9±0.8	30.5±1.0	21.4±0.3
9cis-16:1	17.0±6.1	8.4±3.6	21.4±0.7	7.3±0.1	3.7±0.0
18:0	21.4±0.6	23.1±9.4	21.4±0.7	20.1±0.1	23.4±0.7
9trans 18:1	n.d.	n.d.	n.d.	n.d.	n.d.
8cis-18:1	5.3±0.6	7.0±1.7	4.1±1.2	6.3±0.7	6.8±0.8
9cis-18:1	20.1±3.0	35.0±3.0	20.0±0.6	20.9±0.7	18.7±1.0
11cis-18:1	3.7±0.5	5.8±0.6	3.1±1.1	2.0±0.3	1.6±0.2
5cis,8cis-18:2	2.2±0.4	2.4±0.2	2.3±0.7	2.0±0.2	2.6±0.9
18:2 ω 6	6.9±3.1	5.8±0.5	3.2±0.5	4.5±0.2	3.7±0.9
20:0	n.d.	n.d.	n.d.	n.d.	15.8±4.9
20:3 ω6	n.d.	n.d.	n.d.	n.d.	n.d.
20:4 ω6	n.d.	n.d.	n.d.	n.d.	n.d.
20:5 ω3	n.d.	n.d.	n.d.	n.d.	n.d.
22:0	n.d.	n.d.	n.d.	n.d.	n.d.
22:5 ω3	n.d.	n.d.	n.d.	n.d.	n.d.
22:6 ω3	n.d.	n.d.	n.d.	n.d.	n.d.
SFA	63.2±31.6	77.6±8.8	89.9±1.3	87.4±0.5	101.2±1.8
MUFA	36.9±18.5	75.2±8.8	65.6±1.6	67.0±0.3	52.2±0.7
PUFA	7.3±4.7	8.3±0.6	5.5±0.3	6.5±0.3	6.3±0.1
ω6	5.6±3.9	5.8±0.5	3.2±0.5	4.5±0.2	3.7±0.9
ω3	n.d.	n.d.	n.d.	n.d.	n.d.
Total *trans*	n.d.	n.d.	0.1±0.1	0.2±0.2	1.3±12

[1]FAME identified by the standard references and quantified as described in Materials and Methods; the values are obtained from the main GC peak areas (>97% of the total peak areas of the chromatogram); nd = not detected.

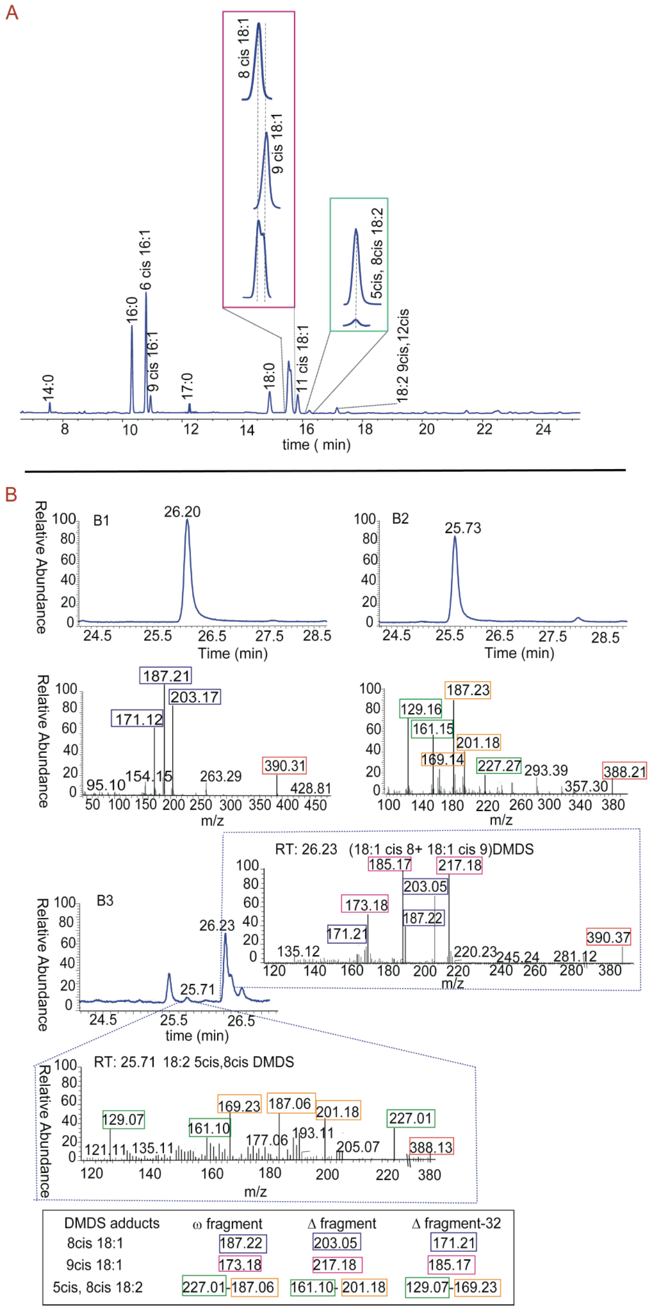

Figure S2. (A) Representative GC analysis of the fatty acid methyl esters (FAME) of cell membrane phospholipids (at 24 h incubation with 150 μM sapienic acid) evidencing the relevant fatty acids discussed in the main text; in the purple box: the enlargement of the chromatogram with the peaks of 8*cis*-18:1 and 9*cis*-18:1 as reference standards and as present in the representative cell sample; in the green box the PUFA formed de novo 5*cis*,8*cis*-18:2 during the sapienic acid metabolism in comparison with the standard reference (upper trace). (B) Representative GC-MS of FAME resulting from the cell culture experiments of supplementation, after treatment with the DMDS derivatization protocol as described in Materials and Methods. GC traces and corresponding mass spectra of the

DMDS adducts of fatty acid methyl esters: (B1) DMDS adduct of the standard reference 8*cis*-18:1; (B2) DMDS adduct of the standard reference of 5*cis*,8*cis*-18:2 and (B3) DMDS adducts of FAME mixture obtained from membrane phospholipids of Caco-2 cell line after sapienic acid supplementation; in the box the diagnostic fragmentation peaks of the DMDS adducts recognized in the sample are reported.
